# Papel do Jejum Intermitente e da Dieta Restrita em Carboidratos na Prevenção de Doenças Cardiovasculares em Pacientes Pré-Diabéticos

**DOI:** 10.36660/abc.20220606

**Published:** 2023-03-27

**Authors:** Mohamed Khalfallah, Basma Elnagar, Shaimaa S. Soliman, Ahmad Eissa, Amany Allaithy

**Affiliations:** 1 Tanta University Faculty of Medicine Cardiovascular Department Egito Cardiovascular Department, Faculty of Medicine, Tanta University – Egito; 2 Menoufia University Faculty of Medicine Public Health and Community Medicine Department Egito Public Health and Community Medicine Department, Faculty of Medicine, Menoufia University – Egito; 3 Tanta University Faculty of Medicine Internal Medicine Department Egito Endocrinology, Internal Medicine Department, Faculty of Medicine, Tanta University – Egito

**Keywords:** Estado Pré-diabético, Jejum, Dieta, Doenças Cardiovasculares

## Abstract

**Fundamentos:**

Pacientes pré-diabéticos têm alto risco de doenças cardiovasculares e complicações microvasculares e macrovasculares. O Jejum Intermitente (JI) e a dieta restrita em carboidratos (dieta low-carb, DLC) são estratégias dietéticas promissoras nesse grupo.

**Objetivos:**

Analisar os benefícios da combinação do JI com DLC sobre desfechos microvasculares e macrovasculares em pacientes pré-diabéticos.

**Métodos:**

O estudo incluiu 485 pacientes pré-diabéticos sem história de doença cardiovascular. Os pacientes foram divididos em dois grupos: grupo I (n = 240) submetidos ao JI (16 horas de JI, F 3-4 dias por semana) combinado com DLC (<130 g de carboidratos por dia), e grupo II (n = 245) que consumiram alimentos à vontade (grupo controle). Os dois grupos foram acompanhados por dois anos para avaliação de complicações macrovasculares e microvasculares. Um valor p < 0,05 foi considerado estatisticamente significativo.

**Resultados:**

Houve uma redução significativa no peso corporal, índice de massa corporal, porcentagem de gordura corporal e hemoglobina glicada no grupo I. A incidência de progressão de pré-diabetes para diabetes foi significativamente menor no grupo I (2,1%) que no grupo II (6,9%) (p = 0,010). Ainda, um aumento significativo na incidência de complicações microvasculares e macrovasculares foi observado no grupo II, incluindo retinopatia, neuropatia e angina instável. A análise de regressão multivariada revelou que peso corporal aumentado, e níveis elevados de glicemia de jejum, hemoglobina glicada e lipoproteína de baixa densidade foram fatores de risco independentes de desfechos microvasculares e macrovasculares.

**Conclusões:**

Em pacientes pré-diabéticos, o JI, combinado com DLC, associou-se com menor progressão para diabetes mellitus e menor incidência de complicações microvasculares e macrovasculares.

**Figure:**
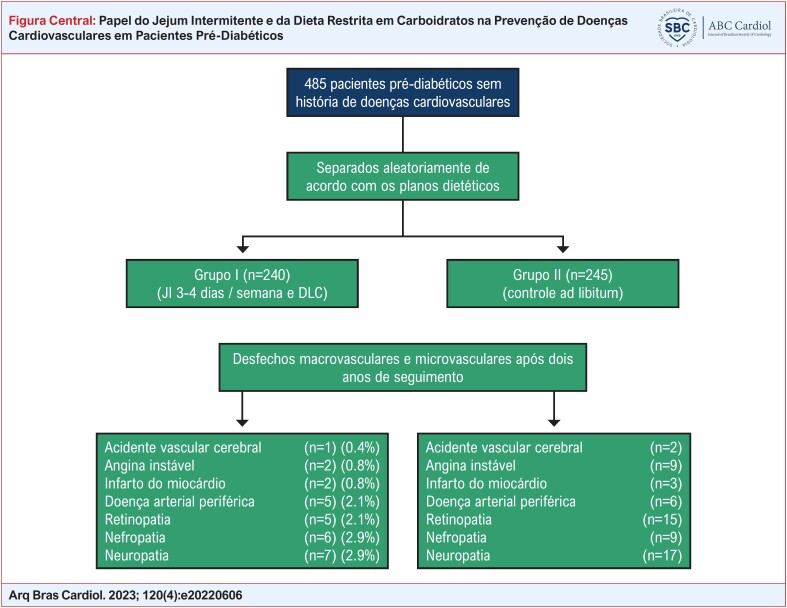
Principais resultados do estudo; JI: jejum intermitente; DLC: dieta restrita em carboidratos (dieta low-carb).

## Introdução

As doenças cardiovasculares (DCVs) ainda são causa importante de morte em todo o mundo, sendo responsáveis por aproximadamente 20% das mortes.^1^ Apesar dos avanços no tratamento das DCVs, sua morbidade e sua mortalidade são significativamente altas, que levam a encargos econômicos. Assim, a detecção e o tratamento precoce dos fatores de risco modificáveis para DCVs são de fundamental importância. Os principais fatores de risco modificáveis incluem sobrepeso, dislipidemia, hipertensão e hiperglicemia.^2^ A pré-diabetes é caracterizada por um estado hiperglicêmico, abaixo do nível utilizado para a definição de diabetes mellitus (DM), com alta probabilidade de progredir para DM. Pacientes pré-diabéticos têm risco elevado de disfunção endotelial, resultando em complicações microvasculares e macrovasculares associadas com maior morbidade e mortalidade cardiovascular.^3^ A incidência de pré-diabetes tem aumentado e está associada à elevação dos níveis de obesidade em todo o mundo.^4^

Vários estudos validaram intervenções no estilo de vida como métodos efetivos de prevenir ou retardar a progressão do DM tipo 2 (DM2) bem como diminuir o risco cardiovascular em pacientes pré-diabéticos.^5,6^ A redução do peso por meio de mudança no estilo de vida, plano alimentar, intervenções farmacológicas e/ou cirúrgicas tem um impacto benéfico sobre o status hiperglicêmico.

O Jejum Intermitente (JI) é um plano dietético caracterizado por uma redução na ingestão energética usando um período de jejum alternando com um consumo livre de alimentos. A forma mais comum consiste em horas de jejum seguidas de horas de ingestão livre em um mesmo dia.^7^ Outra intervenção dietética, principalmente no estado hiperglicêmico, é conhecida como dieta com restrição de carboidratos, ou dieta *low-carb* (DLC). De acordo com o consumo de carboidratos e sua porcentagem do total de energia ingerida, a DLC é classificada como muito baixa em carboidratos (20-50 g/dia ou <10% da ingestão energética), baixa em carboidratos (< 130 g/dia ou < 26% ingestão energética), e moderada em carboidratos (26-44% da ingestão energética).^8^

Nos anos recentes, o JI e as DLCs foram aprovadas e se tornado cada vez mais populares para a redução de peso.^9^ No entanto, seu impacto sobre a saúde humana, especialmente sobre desfechos cardiovasculares em pacientes diabéticos e pré-diabéticos ainda está sob investigação. Este estudo teve como objetivo investigar o impacto do JI e da DLC sobre desfechos microvasculares e macrovasculares em pacientes pré-diabéticos.

### Pacientes e métodos

Este é um estudo prospectivo do tipo coorte, conduzido com 485 participantes atendidos no ambulatório de doenças cardiovasculares do hospital da Universidade de Tanta, Egito, entre junho de 2019 e junho de 2020, com um período de acompanhamento de dois anos. O estudo foi aprovado pelo comitê de ética da faculdade de medicina da Universidade de Tanta, Egito, e conduzido de acordo com os princípios da Declaração de Helsinki II. Todos os pacientes incluídos assinaram um termo de consentimento e um código foi atribuído a cada paciente. Os pacientes incluídos foram randomizados utilizando-se um gerador computadorizado de números aleatórios para a seleção aleatória dos blocos permutados, que possuíam tamanho dois e razão idêntica de alocação. A alocação foi feita de maneira sigilosa, usando envelopes opacos numerados selados, os quais foram abertos após os participantes assinarem o termo de consentimento e serem incluídos nos grupos respectivos.

### Critérios de inclusão e exclusão

Todos os participantes tinham idade acima de 30 anos e abaixo de 70 anos, com diagnóstico confirmado de pré-diabetes por hemoglobina glicada (HbA1c) 5,7-6,4 %, e/ou glicemia de jejum de 100–125 mg/dL, e /ou glicemia duas horas pós-prandial de 140–199 mg/dL.^9^ Pacientes com história prévia de DM, DCVs (acidente vascular cerebral, doença cardíaca isquêmica, insuficiência cardíaca, e doença arterial periférica), doença renal ou hepática avançada, e doença inflamatória crônica foram excluídos.

Os participantes foram separados aleatoriamente em dois grupos de acordo com seu plano dietético; grupo I (JI-DLC): submetido ao JI (16/8: 16 horas de jejum seguido de oito horas de ingestão livre por 3-4 dias por semana), e DLC (menos que 130 g/dia de carboidrato e < 26% da ingestão energética total, sem aumento da ingestão de gordura por sete dias da semana); grupo II (grupo controle): os pacientes continuaram seu padrão dietético diário, sem restrição energética. Todos os pacientes foram submetidos a uma anamnese detalhada, envolvendo fatores de risco cardiovasculares – tabagismo, hipertensão, dislipidemia, história familiar de doenças cardiovasculares – e manifestações de complicações vasculares do DM (por exemplo, dor torácica, visão embaçada e dormência). Estresse psicossocial em todos os participantes foi estimado usando a escala de estresse percebido de 0 a 40 (escore < 14 indicam ausência ou baixo estresse), e escores ≥ 14 indicam status de estresse considerável.^10,11^ A atividade física dos participantes foi avaliada de acordo com o tipo e a duração da atividade física semanal; participantes que realizavam pelo menos 150 minutos de atividade física moderada, ou pelo menos 75 minutos de atividade física vigorosa foram considerados fisicamente ativos.^12^ Os participantes também foram interrogados quanto a seguro saúde, estresse psicossocial, e uso e adesão a medicamentos anti-hipertensivos, hipolipemiantes e antiplaquetários. Ainda, foram avaliados status socioeconômico incluindo nível educacional, renda dos pacientes, local de residência, estado civil e de emprego.

Medidas antropométricas e exames físicos foram realizados, incluindo medida da altura, circunferência da cintura, circunferência do quadril, e razão cintura-quadril usando uma fita métrica. O peso corporal, o índice de massa corporal (IMC) e a composição corporal por análise de bioimpedância elétrica foram aferidos por um aparelho InBody230. Medidas de pressão arterial e frequência cardíaca, e avaliação neurológica completa também foram realizadas. Medidas laboratoriais de rotina incluindo glicemia (jejum e duas horas pós-prandial), colesterol total, lipoproteína de baixa densidade (LDL), lipoproteína de alta densidade (HDL), proteína C reativa, creatinina sérica e taxa de filtração glomerular estimada (TFGe) também foram analisadas. Uma amostra de urina foi coletada pela manhã para análise de albuminúria e razão albumina/creatinina. Ainda, o índice tornozelo-braquial é calculado pela razão entre a pressão arterial sistólica mais alta do tornozelo e a pressão arterial sistólica mais alta da artéria braquial. A espessura da camada íntima média da artéria carótida foi obtida por ultrassonografia das artérias carótidas. Um exame de fundoscopia foi usado para rastrear retinopatia diabética. O escore de risco Framingham foi usado para estimar risco cardiovascular em 10 anos. O risco em 10 anos, em porcentagem, foi classificado como baixo risco (<10%), risco intermediário (10-20%), e alto risco (>20%).^13^ Todas as avaliações clínicas foram realizadas no basal e no seguimento. Durante o acompanhamento de dois anos, os participantes de ambos os grupos fizeram visitas regulares a cada três meses no ambulatório para revisão da planilha mensal da dieta e avaliação da adesão ao JI e DLC (grupo I). Os participantes que perderam uma visita ou não eram aderented ao JI ou à DLC prescrita foram excluídos do estudo.

#### Desfechos

Os desfechos deste estudo foram divididos em desfechos primários, que incluíram mortalidade e ocorrência de complicações macrovasculares como acidente vascular cerebral, angina instável, infarto do miocárdio, e progressão para DM. Os desfechos secundários foram ocorrência de complicações microvasculares: (1) retinopatia, definida como fraqueza progressiva dos vasos da retina, variando de retinopatia não proliferativa e pré-proliferativa à retinopatia proliferativa;^14^ (2) nefropatia, caracterizada por um declínio progressivo na TFGe abaixo de 90mL/min/1,73m^2^, albuminúria persistente, e pressão arterial elevada;^15^ e (3) neuropatia, diagnosticada clinicamente, com várias manifestações clínicas na forma de perda ou anormalidade da função motora, sensorial e/ou do sistema nervoso autônomo.^14^

### Análise estatística

A análise estatística foi realizada usando o programa SPSS versão 23 (Armonk, NY; IBM Corp.). Quanto à normalidade dos dados, usamos o teorema central do limite, que afirma que, quando o tamanho da amostra for maior ou igual a 100, a violação da normalidade não é uma questão importante.^16^ As variáveis quantitativas foram expressas em média ± desvio padrão. As variáveis qualitativas foram expressas em frequência e porcentagem. Usamos o teste t para amostras independentes para comparação das variáveis quantitativas entre os dois grupos. O teste do qui-quadrado (χ^2^) foi usado para comparar dois parâmetros qualitativos. Quando o valor esperado em uma célula foi inferior a cinco, o teste exato de Fisher foi usado. Um valor de p bicaudal <0,05 foi considerado estatisticamente significativo, e análise de regressão logística multivariada foi realizada para detectar preditores independentes de desfechos macrovasculares e microvasculares.

## Resultados

O presente estudo foi realizado com 485 pacientes pré-diabéticos sem evidência de doenças cardiovasculares. Não houve diferença estatisticamente significativa entre os dois grupos quanto à idade, sexo, fatores socioeconômicos, fatores de risco cardiovasculares, e nível educacional (Tabela 1). Não houve diferença significativa nas medidas antropométricas ou dados laboratoriais entre os dois grupos (Tabela 2).

**Tabela 1:** Características basais, fatores de risco e fatores socioeconômicos dos pacientes submetidos ao jejum intermitente e à dieta restrita em carboidratos (grupo I) e ao consumo ad libitum (controle)

		Grupo I (n=240) (JI e DLC)	Grupo II (n=245) (controle ad libitum)	Valor p
Idade, anos		48,11±7,58	48,64±8,46	0,470
Sexo masculino, n (%)		123 (51,3%)	122 (49,8%)	0,749
Tabagismo, n (%)		73 (30,4%)	68 (27,8%)	0,519
Hipertensão, n (%)		65 (27,1%)	77 (31,4%)	0,293
Dislipidemia, n (%)		80 (33,3%)	78 (31,8%)	0,725
História familiar de DCI, n (%)		61 (25,4%)	64 (26,1%)	0,859
Estresse psicossocial, n (%)		53 (22,1%)	51 (20,8%)	0,734
Sedentarismo, n (%)		86 (35,8%)	90 (36,7%)	0,836
Medicamento anti-hipertensivo hypertensive medication use, n (%)		43 (17,9%)	49 (20,0%)	0,558
Hipolipemiante, n (%)		66 (27,5%)	60 (24,5%)	0,450
Medicamento antiplaquetário, n (%)		22 (9,2%)	21 (8,6%)	0,818
Estado civil				
	Casado, n (%)	153 (63,8%)	158 (64,5%)	0,865
	Separado/Divorciado/ Solteiro/Viúvo, n (%)	87 (36,3%)	87 (35,5%)	
Renda				
	Alta, n (%)	139 (57,9%)	129 (52,7%)	0,244
	Baixa, n (%)	101 (42,1%)	116 (47,3%)	
Escolaridade				
	Superior, n (%)	124 (51,7%)	112 (45,7%)	0,190
	Médio ou inferior, n (%)	116 (48,3%)	133 (54,3%)	
Moradia,				
	Urbana, n (%)	133 (55,4%)	139 (56,7%)	0,770
	Rural, n (%)	107 (44,6%)	106 (43,3%)	
Status empregatício,				
	Empegado, n (%)	138 (57,5%)	147 (60,0%)	0,576
	Desempregado, n (%)	102 (42,5%)	98 (40,0%)	
Seguro saúde, n (%)		124 (51,7%)	128 (52,2%)	0,899
Escore de risco Framingham (%)		8,46 ± 6,98	8,71 ± 6,76	0,682

JI: jejum intermitente; DCI: doença cardíaca isquêmica; DLC: dieta restrita em carboidratos (dieta low-carb).

**Tabela 2:** Medidas antropométricas e dados laboratoriais basais dos pacientes submetidos ao jejum intermitente e à dieta restrita em carboidratos (grupo I) e ao consumo ad libitum (controle)

	Grupo I (n=240) (JI e DLC)	Grupo II (n=245) (controle ad libitum)	Valor p
Peso (Kg)	73,52 ± 14,28	73,71 ± 11,94	0,869
Altura (cm)	1,67 ± 0,10	1,66 ± 0,08	0,563
IMC, (kg/m^2^)	26,44 ± 4,90	26,72 ± 3,45	0,470
Circunferência da cintura (cm)	98,72 ± 9,64	99,09 ± 7,04	0,626
Circunferência do quadril (cm)	103,5 ± 7,96	103,6 ± 7,09	0,905
Razão cintura-quadril	0,952 ± 0,04	0,955 ± 0,02	0,203
Gordura corporal (%)	27,11 ± 5,31	26,67 ± 4,41	0,318
Músculo esquelético (%)	28,28 ± 2,13	28,06 ± 1,42	0,166
Gordura visceral (%)	16,27 ± 0,46	16,32 ± 0,51	0,275
PA sistólica, mmHg	133,0 ± 13,1	131,8 ± 12,1	0,303
PA diastólica, mmHg	78,39 ± 10,5	77,71 ± 10,0	0,467
Frequência cardíaca (bpm)	75,3 ± 13,2	77,33 ± 11,8	0,091
FEVE, (%)	62,45 ± 3,36	62,88 ± 4,20	0,213
Glicemia de jejum (mg/dL)	114,9 ± 3,07	115,2 ± 2,78	0,208
Glicemia 2-h pós-prandial (mg/dL) (mmol/L)	164,4 ± 14,8	162,8 ± 17,7	0,288
HbA1c %	5,98 ± 0,24	6,01 ± 0,23	0,306
Hemoglobina, g/dL	12,39 ± 0,73	12,33 ± 0,74	0,429
Colesterol total (mg/dL)	216,9 ± 38,8	213,0 ± 37,3	0,262
TG (mg/dL)	156,8 ± 18,5	154,6 ± 11,1	0,109
LDL (mg/dL)	134,1 ± 24,5	136,6 ± 25,5	0,277
HDL (mg/dL)	44,7 ± 6,90	45,1 ± 7,32	0,502
Creatinina sérica (mg/dL)	1,03 ± 0,21	1,02 ± 0,14	0,980
TFGe (mL/min/1,73 m^2^)	94,1 ± 17,9	93,2 ± 10,6	0,515
Albuminúria (mg/g)	29,14 ± 4,82	28,88 ± 5,01	0,561
PCR (mg/L)	3,55 ± 1,66	3,67 ± 1,35	0,384
Ácido úrico (mg/dL)	5,78 ± 1,19	5,84 ± 0,57	0,494
Troponina I sérica (ng/mL)	0,031 ± 0,01	0,031 ± 0,02	0,740
Índice tornozelo-braquial	1,07 ± 0,15	1,04 ± 0,14	0,083
EIM carótida (mm)	0,97 ± 0,11	0,99 ± 0,10	0,103

JI: jejum intermitente; IMC: índice de massa corporal; PA: pressão arterial; FEVE: fração de ejeção do ventrículo esquerdo; TG: triglicerídeos, LDL: lipoproteína de baixa densidade; HDL: lipoproteína de alta densidade; TFGe: taxa de filtração glomerular estimada; PCR: proteína c reativa; HbA1c: hemoglobina glicada; EIM: espessura da camada íntima média da artéria carótida; DLC: dieta restrita em carboidratos (dieta low-carb).

Após dois anos de seguimento, os seguintes parâmetros foram significativamente mais baixos no grupo I que no grupo II: peso corporal, IMC, circunferência da cintura, e porcentagem de gordura corporal. A porcentagem de redução foi de 5,3% no peso corporal, 5,67 % no IMC, 1,12% na circunferência da cintura, e 6,6% na porcentagem de gordura. No entanto, no grupo II, houve aumento nesses parâmetros em 1,6%, 1,7%, 0.2%, e 1,8% respectivamente, sem diferença significativa entre os dois grupos quanto à gordura visceral ou porcentagem de músculo esquelético. As mudanças na pressão arterial sistólica, diastólica e frequência cardíaca não foram estatisticamente significativas. A glicemia de jejum e a HbA1c% foram estatisticamente mais altas no grupo II que no grupo I; contudo, não houve diferença estatisticamente significativa na glicemia de duas horas pós-prandial entre os grupos. Ainda, o número de pacientes que mostraram progressão de pré-diabetes para DM foi maior no grupo II que no grupo I.

**Figura 1:**
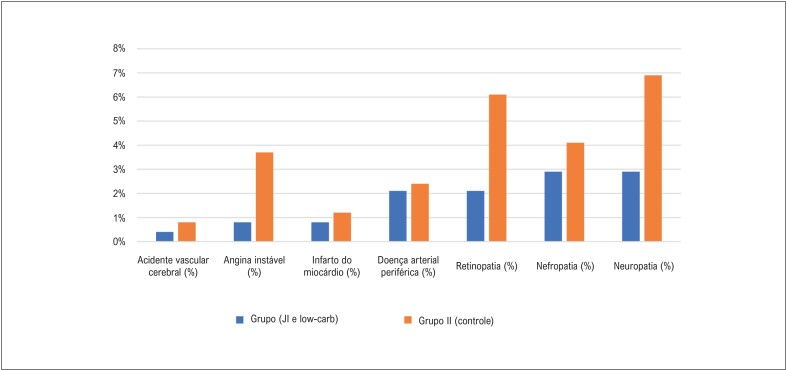
Desfechos macrovascular e microvascular de pacientes submetidos ao jejum intermitente (JI) e dieta restrita em carboidratos (low-carb) (Grupo I) ou consumo ad libitum (Grupo II, controle) após dois anos de seguimento.

Quanto ao perfil lipídico, a única alteração foi a redução significativa nos níveis de LDL no grupo I em comparação ao grupo II. A albuminúria foi significativamente maior no grupo II que no grupo I, enquanto a creatinina e a TFGe não foram significativamente diferentes entre os dois grupos. Ainda, não houve diferença em proteína C reativa, ácido úrico, ou troponina sérica I entre os dois grupos (Tabela 3). Desfechos microvasculares e macrovasculares ocorreram com menor frequência no grupo I, uma frequência significativamente maior de retinopatia, neuropatia, e angina instável foi observada no grupo II que no grupo I (Tabela 4 e Figura 1). Análise de regressão multivariada foi realizada para identificar fatores que afetassem desfechos microvasculares e macrovasculares, e revelou que peso corporal, glicemia de jejum, HbA1c% e LDL aumentados eram os preditores independentes de desfechos microvasculares e macrovasculares, como mostrado na Tabela 5.

**Tabela 3:** Medidas antropométricas e dados laboratoriais basais dos pacientes submetidos ao jejum intermitente e à dieta restrita em carboidratos (grupo I) e ao consumo ad libitum (controle) após dois anos de acompanhamento

	Grupo I (n=240) (JI e DLC)	Grupo II (n=245) (controle ad libitum)	Valor p
Peso (Kg)	69,60 ± 14,75	74,90 ± 12,80	0,001*
Altura (cm)	1,67 ± 0,10	1,66 ± 0,08	0,563
IMC, (kg/m^2^)	24,94 ± 4,76	27,17 ± 3,60	0,001*
Circunferência da cintura (cm)	97,61 ± 10,8	99,31 ± 7,77	0,047*
Circunferência do quadril (cm)	103,0± 8,20	104,2 ± 7,01	0,094
Razão cintura-quadril	0,949 ± 0,04	0,954 ± 0,02	0,087
Gordura corporal (%)	25,32 ± 4,20	27,15 ± 4,48	0,001*
Músculo esquelético (%)	27,93 ± 2,21	28,14 ± 1,39	0,209
Gordura visceral (%)	16,27 ± 0,47	16,35 ± 0,51	0,073
PA sistólica, mmHg	134,7 ± 12,2	133,5 ± 10,6	0,234
PA diastólica, mmHg	78,64 ± 10,2	77,15 ± 9,86	0,105
Frequência cardíaca (bpm)	75,68 ± 13,1	77,47 ± 13,1	0,136
FEVE, (%)	62,78 ± 3,65	63,24 ± 4,32	0,209
Glicemia de jejum (mg/dL)	113,4 ± 3,18	118,1 ± 5,04	0,001*
Glicemia 2-h pós-prandial (mg/dL) (mmol/L)	161,9 ± 15,3	164,8 ± 17,7	0,052
HbA1c %	5,89 ± 0,23	6,24 ± 0,24	0,001*
Progressão para diabetes mellitus	5 (2,1%)	17 (6,9%)	0,010*
Hemoglobina, g/dL	12,37 ± 0,73	12,30 ± 0,75	0,321
Colesterol total (mg/dL)	210,9 ± 39,2	216,9 ± 37,9	0,087
TG (mg/dL)	153,3 ± 31,6	155,1 ± 11,3	0,390
LDL (mg/dL)	132,1 ± 24,2	139,1 ± 25,7	0,002*
HDL (mg/dL)	44,07 ± 6,76	44,86 ± 7,36	0,219
Creatinina sérica (mg/dL)	1,04± 0,21	1,07 ± 0,16	0,165
TFGe (mL/min/1,73 m^2^)	93,01 ± 17,7	91,95 ± 10,0	0,415
Albuminúria (mg/g)	28,75 ± 4,69	30,33 ± 5,19	0,001*
PCR (mg/L)	3,59 ± 1,65	3,71 ± 1,37	0,388
Ácido úrico (mg/dL)	5,98 ± 0,78	5,87 ± 0,57	0,088
Troponina I sérica (ng/mL)	0,030 ± 0,01	0,032± 0,01	0,111
Índice tornozelo-braquial	1,05 ± 0,15	1,08 ± 0,14	0,073
EIM carótida (mm)	0,99 ± 0,11	1,01 ± 0,10	0,078

* valor p significativo; JI: jejum intermitente; IMC: índice de massa corporal; PA: pressão arterial; FEVE: fração de ejeção do ventrículo esquerdo; TG: triglicerídeos; LDL: lipoproteína de baixa densidade; HDL: lipoproteína de alta densidade; TFGe: taxa de filtração glomerular estimada; PCR: proteína c reativa; HbA1c: hemoglobina glicada; EIM: espessura da camada íntima média da artéria carótida; DLC: dieta restrita em carboidratos (dieta low-carb).

**Tabela 4:** Desfechos macrovasculares e microvasculares em pacientes submetidos ao jejum intermitente e à dieta restrita em carboidratos (grupo I) e ao consumo ad libitum (controle) após dois anos de acompanhamento

	Grupo I (n=240) (JI e DLC)	Grupo II (n=245) (controle ad libitum)	Valor p
Mortalidade, n (%)	0 (0%)	0 (0%)	
Acidente vascular cerebral, n (%)	1 (0,4%)	2 (0,8%)	0,575
Angina instável, n (%)	2 (0,8%)	9 (3,7%)	0,036*
Infarto do miocárdio, n (%)	2 (0,8%)	3 (1,2%)	0,670
Doença arterial periférica, n (%)	5 (2,1%)	6 (2,4%)	0,787
Retinopatia, n (%)	5 (2,1%)	15 (6,1%)	0,025*
Nefropatia, n (%)	6 (2,9%)	9 (4,1%)	0,455
Neuropatia, n (%)	7 (2,9%)	17 (6,9%)	0,041*

* valor p significativo. JI: jejum intermitente; DLC: dieta restrita em carboidratos (dieta low-carb).

**Tabela 5:** Análise de regressão multivariada mostrando os fatores de risco independentes dos desfechos microvasculares e macrovasculares

	Análise multivariada		Valor p
	OR	(IC95%)	
Peso corporal	1,056	1,023– 1,090	0,001*
Índice de massa corporal	1,069	0,928– 1,230	0,356
Circunferência da cintura	1,024	0,975– 1,076	0,348
Gordura corporal %	1,068	0,983– 1,161	0,122
Glicemia de jejum	1,100	1,027– 1,178	0,006*
HbA1c %	9,575	2,175– 42,157	0,003*
LDL	1,078	1,039– 1,120	0,001*
Albuminúria	1,071	0,966– 1,187	0,190

LDL: lipoproteína de baixa densidade. HbA1c: hemoglobina glicada.

## Discussão

Os pacientes pré-diabéticos apresentam maior risco para DCVs e suas complicações macrovasculares e microvasculares. Mudanças no estilo de vida, incluindo dieta, atividade física, e cessação do tabagismo têm um papel crucial no manejo do pré-diabetes e do diabetes e redução de complicações. A dieta exerce um papel essencial no manejo em geral, incluindo nas complicações cardiovasculares.^17^ Assim, no presente estudo, investigamos o valor de se combinar duas estratégias dietéticas (JI e DLC) na prevenção de complicações microvasculares e macrovasculares em pacientes pré-diabéticos.

Neste estudo, encontramos uma redução significativamente maior no peso corporal, IMC, circunferência da cintura, e porcentagem de gordura no grupo I em comparação ao grupo II, mas não houve diferença significativa na gordura visceral ou porcentagem de músculo esquelético entre os grupos. Esses resultados estão de acordo com os apresentados por Kalam et al.,^18^ que estudaram 31 pacientes obesos submetidos a uma combinação de JI e DLC durante seis meses, e demonstraram reduções significativas no peso corporal (em 6,3±1,0%) e massa de gordura (p<0,01), e nenhuma alteração significativa na gordura visceral ou na massa magra. Ainda, O’Driscoll et al.,^19^ combinaram JI e DLC por 12 meses e relataram uma redução significativa no peso corporal em 9%, circunferência da cintura, e IMC em 8,6%. Moro et al.^20^ estudaram o efeito do JI em homens sadios treinados e encontraram uma diminuição na massa de gordura em 16,4% no grupo JI em comparação a uma diminuição em 2,8% no grupo controle, enquanto não houve alteração na massa livre de gordura e na massa muscular esquelética em ambos os grupos. Por outro lado, Zaki et al.^21^ mostraram que uma DLC pode reduzir o peso corporal, mas de modo menos eficaz que a dieta cetogênica.

Ainda, o grupo I apresentou melhora no perfil glicêmico por meio da redução significativa na glicemia de jejum e HbA1c. Wilkinson et al.^22^ e Wei et al.^23^ mostraram melhora semelhante na HbA1c e glicemia de jejum pelo JI, principalmente nos indivíduos que apresentavam uma glicemia de jejum basal mais alta. Ainda, Yamada et al.^24^ relataram uma redução significativa nos níveis de HgA1c (7,6 ± 0,4% vs. 7,0 ± 0,7%) nos indivíduos com DM2 submetidos à DLC em comparação àqueles submetidos à restrição calórica (7,7 ± 0,6% vs. 7,5 ± 1,0%). Contudo, não houve diferença significativa no peso corporal ou IMC. No estudo de Kalam et al.,^18^ embora não houve alteração na glicemia de jejum e na HbA1c após seis meses de DLC e JI, observou-se uma redução na insulina de jejum em 24%, o que pode ser explicado pelo curto período de intervenção com o JI.

Além disso, o presente estudo mostrou que a progressão de pré-diabetes para DM2 foi estatisticamente maior no grupo controle (6,9%) que no grupo JI-DLC. Esse dado está de acordo com o estudo de Wang et al.,^25^ que descreveram uma redução significativa na glicemia de jejum e na glicemia pós-prandial, acompanhada por uma redução na dose de insulina em 8,7% nos pacientes com DM2 submetidos à DLC por três meses. Em uma meta-análise comparando a DLC com a dieta hipolipídica em pacientes com DM2, os pacientes submetidos à DLC atingiram maiores taxas de remissão do DM em seis meses.^26^ Tal fato foi explicado por Sutton et al.,^27^ que avaliaram o efeito do JI em pacientes pré-diabéticos, e relataram um aumento na sensibilidade da insulina e função das células-beta, principalmente nos pacientes com sobrepeso. Furmli et al.^28^ também confirmaram uma redução na necessidade de insulina em pacientes com DM2 com o uso do JI.

Outro resultado importante do presente estudo foi a redução significativamente maior nos níveis de LDL no grupo I em comparação ao grupo II, sem alteração nos níveis de colesterol total, triglicerídeos ou HDL. Esse achado foi similar aos estudos de Kalam et al.^18^ e Jacobi et al.,^29^ que descreveram uma redução significativa no LDL e no colesterol total, e ausência de alteração nos níveis de triglicerídeos e HDL. Por outro lado, quatro artigos comparando JI e consumo *ad libitum* relataram uma redução no colesterol total, sem alteração no LDL, triglicerídeos ou HDL.^30^ Wilkinson et al.,^22^ contudo, relataram uma diminuição, do basal, no colesterol total, LDL, e colesterol não HDL com o JI, sem alteração nos níveis de triglicerídeos ou HDL. Ainda, o impacto da DLC foi investigado e comparado com uma dieta rica em carboidratos, sem mudanças significativas no perfil lipídico e uma leve redução nos níveis de triglicerídeos.^31^ O presente estudo mostrou uma mudança significativa na albuminúria, sem alteração na creatinina sérica, TFGe, e incidência de nefropatia. Sulaj et al.^32^ também mostraram que o JI foi mais efetivo em melhorar a albuminúria em comparação à dieta mediterrânea, sem diferença na TFGe ou creatinina sérica entre os dois grupos.

Assim, em relação aos desfechos primários, a incidência de angina instável foi significativamente maior no grupo I que nos controles. Esses resultados são reforçados pela elevação significativa na HbA1c no grupo II, bem como pela elevação no LDL, precursor de aterosclerose. De fato, Focardi et al.^33^ demonstraram uma melhora na função endotelial pela DLC. Vários estudos relataram o impacto do JI e da DLC sobre a morbidade e mortalidade por DCV pela melhora no perfil lipídico e glicêmico, além da mudança nos fatores de risco. Ainda, a incidência de retinopatia e neuropatia foi significativamente menor no grupo I (2,1% e 2.9 %, respectivamente) que no grupo II (6,1% e 6,9%, respectivamente).^34–36^ Esses desfechos comprovam o impacto do JI e da DLC na diminuição das complicações microvasculares. Nossos dados vão ao encontro dos descritos por Hammer et al.^37^ e Dannawi et al.,^38^ que revelaram uma redução na retinopatia e neuropatia pelo JI, e Hwang et al.^39^ que revelaram uma recuperação na função endotelial microvascular pela DLC somente após seis semanas. Uma baixa incidência de retinopatia e neuropatia está associada com uma redução na HbA1c e glicemia de jejum.^40,41^

## Conclusão

O JI, combinado com a DLC, pode exercer um importante papel na prevenção e no tratamento da DCV em pacientes pré-diabéticos. Este estudo mostrou melhora no status glicêmico, redução na progressão do diabetes e diminuição significativa na incidência de retinopatia, neuropatia, e angina instável no grupo JI-DLC. Ainda, a combinação do JI com a DLC associou-se com melhora nos desfechos primários e secundários em pacientes pré-diabéticos, com redução na morbidade cardiovascular. Aumento no peso corporal e níveis de glicemia de jejum, HbA1c, e LDL elevados foram fatores de risco independentes de desfechos microvasculares e macrovasculares.
