# Supplementary material for: Papel do Jejum Intermitente e da Dieta Restrita em Carboidratos na Prevenção de Doenças Cardiovasculares em Pacientes Pré-Diabéticos
Source: Arq Bras Cardiol. 2023 Mar 27;120(4):e20220606. [Article in Portuguese] doi: 10.36660/abc.20220606 (PMC10263423; doi:10.36660/abc.20220606)
Supplement: Translation

# Translation: The Value of Intermittent Fasting and Low Carbohydrate Diet in Prediabetic Patients for the Prevention of Cardiovascular Diseases

**Authors:** Mohamed Khalfallah, Basma Elnagar, Shaimaa S. Soliman, Ahmad Eissa, Amany Allaithy

**Affiliations:** 1 Egypt Cardiovascular Department, Faculty of Medicine, Tanta University – Egypt; 2 Egypt Public Health and Community Medicine Department, Faculty of Medicine, Menoufia University – Egypt; 6 Egypt Endocrinology, Internal Medicine Department, Faculty of Medicine, Tanta University – Egypt

**Keywords:** Prediabetic State, Fasting, Diet, Cardiovascular Diseases

## Abstract

**Background:**

Prediabetic patients are at increased risk for cardiovascular diseases and the development of microvascular and macrovascular complications. Intermittent fasting (IF) and low-carbohydrate diet (LCD) are promising dietary plans.

**Objectives:**

Our aims to analyze the benefits of IF combined with LCD on microvascular and macrovascular outcomes in prediabetic patients.

**Methods:**

The study included 485 prediabetic patients with no history of cardiovascular diseases divided into group I: (n = 240 patients) who underwent IF (16 h IF 3-4 days per week) combined with LCD (<130 g of carbohydrate per day), and group II: (n = 245 patients) with ad libitum calorie intake. The two groups were followed-up for two years for assessment of micro and macrovascular complications. A p-value < 0.05 was considered statistically significant.

**Result:**

There was a significant reduction in body weight, body mass index, waist circumference, body fat percentage and glycated hemoglobin in group I. The incidence of progression from prediabetes to diabetes was significantly lower in group I (2.1% vs. 6.9% in group II, p = 0.010). In addition, a significant increase in the incidence of microvascular and macrovascular complications was observed in group II, including retinopathy, neuropathy and unstable angina. Multivariate regression analysis revealed that increased body weight, fasting glucose, glycated hemoglobin and low-density lipoprotein were independent risk factors impacting microvascular and macrovascular outcomes.

**Conclusions:**

In prediabetic patients, IF, combined with LCD, was associated with lower progression to diabetes mellitus and lower incidence of microvascular and macrovascular complications.

**Figure:** Central Illustration - Illustration of the main results of the article; IF: intermittent fasting

## Introduction

Cardiovascular diseases (CVD) are still a fundamental cause of death worldwide, accounting for approximately 20% of all deaths.^1^ Despite advances in CVD management, the morbidity and mortality due to CVD are significantly high which lead to an economic burden. Therefore, the early detection and treatment of the modifiable risk factors for CVD are of paramount importance. Major modifiable risk factors include overweight, dyslipidemia, hypertension and hyperglycemia.^2^ Prediabetes is characterized by a hyperglycemic state, below the level used to define diabetes mellitus (DM), with a high probability of progression to DM. Moreover, prediabetic patients are at high risk of endothelial dysfunction resulting in microvascular and macrovascular complications associated with higher cardiovascular morbidity and mortality.^3^ The incidence of prediabetes has increased and been associated with an increase in obesity worldwide.^4^

Several studies have validated lifestyle intervention as an effective method in preventing or delaying the progression to type 2 diabetes mellitus (T2DM) and decreasing the cardiovascular risk in prediabetic patients.^5,6^ Reduction of weight using lifestyle modification, diet plans, pharmacological and/or surgical interventions has a beneficial impact on hyperglycemic status.

Intermittent fasting (IF) is a dietary pattern characterized by a decreased caloric intake using a fasting period alternating with ad libitum food consumption. The most common type consists of hours of fasting followed by hours of free intake on the same day.^7^ Another dietary intervention especially in the hyperglycemic state is known as a low-carb diet (LCD), based on carbohydrate restriction. According to the carbohydrate consumption and its percent of total energy intake, LCD is classified into very LCD (20-50 g/day or <10% of energy intake), LCD (less than 130 g/day or < 26% of energy intake), and moderate carb (26- 44% energy intake).^8^

In recent years, the IF and LCD diets have been approved and become more and more popular and for weight reduction.^9^ However, their impact on human health, especially on cardiovascular outcomes in prediabetic and diabetic patients is still under investigation. This study aimed to investigate the impact of both IF and LCD on microvascular and macrovascular outcomes in prediabetic patients.

### Patients and methods

This is a prospective cohort study, conducted with 485 participants attending the cardiovascular outpatient clinic in Tanta University Hospital, Egypt, from June 2019 to June 2020 with a follow-up period of two years. The study was approved by the research ethics committee of Tanta University Medical School and conducted in accordance with the principles of the Declaration of Helsinki II. All patients included in the study signed a written informed consent form and a code number was assigned to each patient. The included patients were randomized using a computerized random number generator to select randomly permuted blocks with a block size of two and an equal allocation ratio. Allocation concealment was performed using consecutively numbered opaque sealed envelopes which was opened after the patient signed the written consent and then enrolled into the respective group.

### Inclusion and exclusion criteria

All participants were above 30 years old and below 70 years old with a confirmed diagnosis of prediabetes either by glycated hemoglobin (HbA1c) 5.7–6.4% and /or fasting blood glucose of 100–125 mg/dl and/or two-hour post prandial blood glucose of 140–199 mg/dL.^9^ Patients with prior history of DM, CVD (stroke, ischemic heart disease, heart failure, and peripheral arterial disease), advanced liver or renal disease, and chronic inflammatory disease were excluded.

The participants were randomized into two groups according to their dietary plan; group I (IF–LCD): underwent IF (16/8: 16 hours of fasting followed by eight hours of unlimited eating for 3-4 days per week) in addition to low-carb intake (less than 130 g/day of carbohydrate and <26% of total energy intake, without increase in fat intake for seven days a week); group (II) (Control group): control group patients continued their usual daily dietary pattern with no calorie restriction. All participants were submitted to a detailed history taking, including cardiovascular risk factors – smoking, hypertension, dyslipidemia, family history of cardiovascular diseases – and manifestations of vascular complications of DM (e.g. chest pain, blurring of vision, and numbness). Psychosocial stress in all participants was estimated using the perceived stress scale scoring from 0-40 (scores <14 indicate absence or low stress condition, while scores ≥ 14 indicate status of considerable stress).^10,11^ Also, their physical activity was evaluated according to the duration and type of physical activity per week; participants who performed 150 minutes of moderate physical activity or 75 minutes of vigorous physical activity or more per week were considered physically active.^12^ Patients were also asked about health insurance, psychosocial stress, use and compliance to antihypertensive, cholesterol-lowering, and antiplatelet medications, and compliance to these medications. Furthermore, socioeconomic status including educational level, patients’ income, place of residence, marital and employment status, were assessed.

Anthropometric measurements and physical examinations were performed including measurements of height, waist circumference, hip circumference, and waist-hip ratio using a measuring tape. Body weight, body mass index (BMI), and body composition with bioelectrical impedance analysis were measured using the InBody230 device. Blood pressure and heart rate measurements and full neurological evaluation were also performed. Routine laboratory parameters including plasma glucose (fasting, 2 hours postprandial), HbA1c, serum total cholesterol, triglycerides, low-density lipoprotein cholesterol (LDL), high-density lipoprotein cholesterol (HDL), C-reactive protein, serum creatinine and estimated glomerular filtration rate (e-GFR) were also analyzed. A urine sample was taken in the early morning for albuminuria and the albumin/creatinine ratio, and an electrocardiogram and echocardiography were performed in all patients. Furthermore, the ankle-brachial index was calculated by dividing the highest ankle systolic pressure by the highest brachial systolic pressure. Carotid intima-media thickness was obtained by carotid ultrasound. A fundoscopic examination was performed for diabetic retinopathy screening. Framingham risk score was used to estimate 10 years of cardiovascular risk. The ten-year risk in percentage was classified as low risk (< 10%), intermediate risk (10–20%), and high risk (> 20%).^13^ All clinical assessments were performed at baseline and follow-up. During the two years of follow-up, participants of both groups made regular visits every three months at the outpatient clinic for revision of their monthly diet sheet and assessment of the adherence to IF and LCD (group I). The participants who missed a visit or was not adherent to the IF or to the LCD prescribed were excluded from the study.

#### Outcomes

The outcomes of this study were divided into primary endpoints which included mortality and the occurrence of macrovascular complications, such as stroke, unstable angina, and myocardial infarction, and progression to overt DM. Secondary endpoints were the occurrence of microvascular complications: (1) retinopathy, defined as progressive weakness of retinal vessels varying from non-proliferative and pre-proliferative to proliferative retinopathy;^14^ (2) nephropathy, characterized by a progressive decline in e-GFR below 90 mL/min/1.73 m^2^, persistent albuminuria, and elevated arterial blood pressure;^15^ and (3) neuropathy which has many clinical manifestations in the form of abnormal or loss of motor, sensory, and/or autonomic nervous system function, diagnosed clinically.^14^

### Statistical analysis

Statistical analysis was done using SPSS 23, (Armonk, NY; IBM Corp.). As regarding the normality of the data, we used the central limit theorem which states that when the sample size has 100 or more observations, violation of the normality is not a major issue.^16^ Quantitative variables were expressed as mean ± standard deviation. Qualitative variables were expressed as frequency and percentage. Independent samples t-test was used for comparison of quantitative variables between the two groups. The chi-square test (χ2) was used to compare two qualitative parameters. When the expected value in a cell was less than five, the Fisher’s exact test was used. A two-sided p-value <0.05 was considered statistically significant. Multivariate logistic regression analysis was performed to detect the independent predictors of macrovascular and microvascular outcomes.

## Results

The present study was conducted with 485 prediabetic patients with no evidence of cardiovascular diseases. There was no statistically significant difference between the two groups regarding their age, sex distribution, socioeconomic factors, cardiovascular risk factors and level of education, as shown in Table 1. There was no significant difference in baseline anthropometric measurements or laboratory data between the two groups (Table 2).

**Table 1:** Baseline characteristics, risk factors and socioeconomic factors of patients undergoing intermittent fasting (IF) and lowcarbohydrate (low-carb) diet (group I) and ad libitum food intake (control, group II)

		Group I (n=240) (IF and low-carb diet)	Group II (n=245) (Ad libitum control)	p value
Age, years		48.11±7.58	48.64±8.46	0.470
Male gender, n (%)		123 (51.3%)	122 (49.8%)	0.749
Smoking, n (%)		73 (30.4%)	68 (27.8%)	0.519
Hypertension, n (%)		65 (27.1%)	77 (31.4%)	0.293
Dyslipidemia, n (%)		80 (33.3%)	78 (31.8%)	0.725
Family history of IHD, n (%)		61 (25.4%)	64 (26.1%)	0.859
Psychosocial stress, n (%)		53 (22.1%)	51 (20.8%)	0.734
Physical inactivity, n (%)		86 (35.8%)	90 (36.7%)	0.836
Anti-hypertensive medication, n (%)		43 (17.9%)	49 (20.0%)	0.558
Cholesterol-lowering medication, n (%)		66 (27.5%)	60 (24.5%)	0.450
Anti-platelets medication use, n (%)		22 (9.2%)	21 (8.6%)	0.818
Marital status				
	Married, n (%)	153 (63.8%)	158 (64.5%)	0.865
	Separated/Divorced/ Single/ Widow/Widower, n (%)	87 (36.3%)	87 (35.5%)	
Income category				
	High income, n (%)	139 (57.9%)	129 (52.7%)	0.244
	Low income, n (%)	101 (42.1%)	116 (47.3%)	
Level of education				
	Bachelor’s degree or higher, n (%)	124 (51.7%)	112 (45.7%)	0.190
	High school or less, n (%)	116 (48.3%)	133 (54.3%)	
Residence,				
	Urban, n (%)	133 (55.4%)	139 (56.7%)	0.770
	Rural, n (%)	107 (44.6%)	106 (43.3%)	
Occupational status,				
	Employed, n (%)	138 (57.5%)	147 (60.0%)	0.576
	Unemployed, n (%)	102 (42.5%)	98 (40.0%)	
Health insurance, n (%)		124 (51.7%)	128 (52.2%)	0.899
Framingham risk score (%)		8.46 ± 6.98	8.71 ± 6.76	0.682

IHD: ischemic heart disease.

**Table 2:** Baseline anthropometric measurements and laboratory data of patients undergoing intermittent fasting (IF) and low-carbohydrate (low-carb) diet (group I) and ad libitum food intake (control, group II)

	Group I (n=240) (IF and low-carb diet)	Group II (n=245) (Ad libitum control)	p value
Weight (Kg)	73.52 ± 14.28	73.71 ± 11.94	0.869
Height (cm)	1.67 ± 0.10	1.66 ± 0.08	0.563
BMI, (Kg/m^2^)	26.44 ± 4.90	26.72 ± 3.45	0.470
Waist circumference(cm)	98.72 ± 9.64	99.09 ± 7.04	0.626
Hip circumference (cm)	103.5 ± 7.96	103.6 ± 7.09	0.905
Waist-hip ratio	0.952 ± 0.04	0.955 ± 0.02	0.203
Body fat (%)	27.11 ± 5.31	26.67 ± 4.41	0.318
Skeletal muscle (%)	28.28 ± 2.13	28.06 ± 1.42	0.166
Visceral fat (%)	16.27 ± 0.46	16.32 ± 0.51	0.275
Systolic BP, mmHg	133.0 ± 13.1	131.8 ± 12.1	0.303
Diastolic BP, mmHg	78.39 ± 10.5	77.71 ± 10.0	0.467
Heart rate, (bpm)	75.3 ± 13.2	77.33 ± 11.8	0.091
LVEF, (%)	62.45 ± 3.36	62.88 ± 4.20	0.213
Fasting plasma glucose (mg/dL)	114.9 ± 3.07	115.2 ± 2.78	0.208
2-h post prandial plasma glucose (mg/dl) (mmol/L)	164.4 ± 14.8	162.8 ± 17.7	0.288
HbA1c %	5.98 ± 0.24	6.01 ± 0.23	0.306
Hemoglobin, g/dL	12.39 ± 0.73	12.33 ± 0.74	0.429
Total cholesterol (mg/dl)	216.9 ± 38.8	213.0 ± 37.3	0.262
TG (mg/dL)	156.8 ± 18.5	154.6 ± 11.1	0.109
LDL (mg/dL)	134.1 ± 24.5	136.6 ± 25.5	0.277
HDL (mg/dL)	44.7 ± 6.90	45.1 ± 7.32	0.502
Serum creatinine (mg/dL)	1.03 ± 0.21	1.02 ± 0.14	0.980
e-GFR (mL/min/1.73 m^2^)	94.1 ± 17.9	93.2 ± 10.6	0.515
Albuminuria (mg/g)	29.14 ± 4.82	28.88 ± 5.01	0.561
CRP (mg/L)	3.55 ± 1.66	3.67 ± 1.35	0.384
Uric acid (mg/dL)	5.78 ± 1.19	5.84 ± 0.57	0.494
Serum troponin I (ng/mL)	0.031 ± 0.01	0.031 ± 0.02	0.740
Ankle brachial index	1.07 ± 0.15	1.04 ± 0.14	0.083
Carotid IMT (mm)	0.97 ± 0.11	0.99 ± 0.10	0.103

BMI: body mass index; BP: blood pressure; LVEF: left ventricle ejection fraction; HbA1c: glycated hemoglobin; TG: triglyceride; LDL: low density lipoprotein; HDL: high density lipoprotein; e-GFR: estimated glomerular filtration rate; CRP: C - reactive protein; IMT: intima media thickness; BP: blood pressure.

After two years of follow-up, the following parameters were significantly lower in group I than group II: body weight, BMI, waist circumference, and body fat percentage. The percentage of reduction was -5.3% in body weight, -5.67 % in BMI, -1.12% in waist circumference, and -6.6% in body fat percentage, however in group II, there was an increase in these parameters by +1.6%, +1.68%, +0.22%, and +1.79% respectively with no significant difference between the two groups in visceral fat or skeletal muscle percentage. The changes in systolic and diastolic blood pressure and heart rate were not statistically significant. Fasting plasma glucose and HbA1c % were statistically higher in group II than group I; however, there was no statistically significant difference in 2-h postprandial plasma glucose between the two groups. Moreover, the number of patients who showed a progression from prediabetes to DM was higher in group II than group I.

As regarding lipid profile, the only change was the significant reduction in LDL level in group I than group II. Albuminuria was significantly higher in group II than group I, whereas creatinine and e-GFR showed no significant difference between the two groups. Also, C-reactive protein, uric acid, and serum troponin I were not different between the two groups (Table 3). Microvascular and macrovascular outcomes occurred less frequently in group I, and significantly higher frequency of retinopathy, neuropathy, and unstable angina was found in group II than group I (Table 4 and Figure 1). Multivariate regression analysis was performed to identify factors affecting microvascular and macrovascular outcomes, which revealed that the increase in body weight, fasting plasma glucose, HbA1c % and LDL were independent predictors of microvascular and macrovascular outcomes as shown in Table 5.

**Table 3:** Anthropometric measurements and laboratory data of patients undergoing intermittent fasting (IF) and low-carbohydrate (low-carb) diet (group I) and ad libitum food intake (control, group II) after two years follow-up

	Group I (n=240) (IF and low-carb diet)	Group II (n=245) (ad libitum control)	p value
Weight (kg)	69.60 ± 14.75	74.90 ± 12.80	0.001*
Height (cm)	1.67 ± 0.10	1.66 ± 0.08	0.563
BMI, (kg/m^2^)	24.94 ± 4.76	27.17 ± 3.60	0.001*
Waist circumference(cm)	97.61 ± 10.8	99.31 ± 7.77	0.047*
Hip circumference(cm)	103.0± 8.20	104.2 ± 7.01	0.094
Waist-hip ratio	0.949 ± 0.04	0.954 ± 0.02	0.087
Body fat (%)	25.32 ± 4.20	27.15 ± 4.48	0.001*
Skeletal muscle (%)	27.93 ± 2.21	28.14 ± 1.39	0.209
Visceral fat (%)	16.27 ± 0.47	16.35 ± 0.51	0.073
Systolic BP, mmHg	134.7 ± 12.2	133.5 ± 10.6	0.234
Diastolic BP, mmHg	78.64 ± 10.2	77.15 ± 9.86	0.105
Heart rate, (bpm)	75.68 ± 13.1	77.47 ± 13.1	0.136
LVEF, (%)	62.78 ± 3.65	63.24 ± 4.32	0.209
Fasting plasma glucose (mg/dl)	113.4 ± 3.18	118.1 ± 5.04	0.001*
2-h post prandial plasma glucose (mg/dl) (mmol/L)	161.9 ± 15.3	164.8 ± 17.7	0.052
HbA1c %	5.89 ± 0.23	6.24 ± 0.24	0.001*
Progression to diabetes mellitus, n (%)	5 (2.1%)	17 (6.9%)	0.010*
Hemoglobin, g/dL	12.37 ± 0.73	12.30 ± 0.75	0.321
Total cholesterol (mg/dl)	210.9 ± 39.2	216.9 ± 37.9	0.087
TG (mg/dL)	153.3 ± 31.6	155.1 ± 11.3	0.390
LDL (mg/dL)	132.1 ± 24.2	139.1 ± 25.7	0.002*
HDL (mg/dL)	44.07 ± 6.76	44.86 ± 7.36	0.219
Serum creatinine (mg/dL)	1.04± 0.21	1.07 ± 0.16	0.165
e-GFR (mL/min/1.73 m^2^)	93.01 ± 17.7	91.95 ± 10.0	0.415
Albuminuria (mg/g)	28.75 ± 4.69	30.33 ± 5.19	0.001*
CRP (mg/L)	3.59 ± 1.65	3.71 ± 1.37	0.388
Uric acid (mg/dL)	5.98 ± 0.78	5.87 ± 0.57	0.088
Serum troponin I (ng/mL)	0.030 ± 0.01	0.032± 0.01	0.111
Ankle brachial index	1.05 ± 0.15	1.08 ± 0.14	0.073
Carotid IMT (mm)	0.99 ± 0.11	1.01 ± 0.10	0.078

* Significant P value; BMI: body mass index; BP: blood pressure; LVEF: left ventricle ejection fraction; HbA1c: glycated hemoglobin; TG: triglyceride; LDL: low density lipoprotein; HDL: high density lipoprotein; e-GFR: estimated glomerular filtration rate; CRP: C - reactive protein; IMT: intima media thickness.

**Table 4:** Macrovascular and microvascular outcomes in patients undergoing intermittent fasting (IF) and low-carbohydrate (low-carb) diet (group I) and *ad libitum* food intake (control, group II) after two years of follow-up

	Group I (n=240) (IF and low-carb diet)	Group II (n=245) (Ad libitum control)	p value
Mortality, n (%)	0 (0%)	0 (0%)	
Cerebral stroke, n (%)	1 (0.4%)	2 (0.8%)	0.575
Unstable angina, n (%)	2 (0.8%)	9 (3.7%)	0.036*
Myocardial infarction, n (%)	2 (0.8%)	3 (1.2%)	0.670
Peripheral arterial disease, n (%)	5 (2.1%)	6 (2.4%)	0.787
Retinopathy, n (%)	5 (2.1%)	15 (6.1%)	0.025*
Nephropathy, n (%)	6 (2.9%)	9 (4.1%)	0.455
Neuropathy, n (%)	7 (2.9%)	17 (6.9%)	0.041*

* significant P value.

**Figure 1:** Macrovascular and microvascular outcomes of patients undergoing intermittent fasting (IF) and low-carbohydrat (low-carb) diet (Group I) or ad libitum food consumption (Group II, controls) after two years of follow-up.

**Table 5:** Multivariate regression analysis showing the independent risk factors of microvascular and macrovascular outcomes

	Multivariate analysis		p value
	OR	(95% CI)	
Body weight	1.056	1.023– 1.090	0.001*
Body mass index	1.069	0.928– 1.230	0.356
Waist circumference	1.024	0.975– 1.076	0.348
Body fat %	1.068	0.983– 1.161	0.122
Fasting plasma glucose	1.100	1.027– 1.178	0.006*
HbA1c %	9.575	2.175– 42.157	0.003*
LDL	1.078	1.039– 1.120	0.001*
Albuminuria	1.071	0.966– 1.187	0.190

LDL: low density lipoprotein.

## Discussion

Prediabetic patients are at high risk of CVD and its microvascular and macrovascular complications. Lifestyle modifications including diet, physical activity, and smoking cessation, play a crucial role in the management of prediabetes and diabetes and reduction of complications. The diet plays an essential role in overall management including cardiovascular complications.^17^ Therefore, in the current study, we investigated the adding value of the combination of two dietary methods (IF and LCD) in preventing microvascular and macrovascular complications in prediabetic patients.

In this study, we found that there was a significantly greater reduction in body weight, BMI, waist circumference, and fat percentage in group I than in group II, but no significant difference in visceral fat or skeletal muscle percentage between the two groups. In agreement with our results, Kalam et al.^18^ studied 31 obese patients who underwent a combination of IF with low-carb intervention for six months and demonstrated significant reductions in body weight (by 6.3 ± 1.0%) and fat mass (p < 0.01), while visceral fat and skeletal mass remained unchanged. Also, O’Driscoll et al.^19^ combined IF and LCD for 12 months and reported a significant reduction in body weight by 9%, waist circumference, and BMI by 8.6%. Moro et al.^20^ studied the effect of IF in healthy-trained males and found a decrease in fat mass by 16.4 % in the IF group compared to the normal diet group (2.8%), while the fat-free mass and the skeletal mass remained unchanged in both groups. On the other hand, Zaki et al.^21^ showed that a LCD can reduce body weight but not as effectively as ketogenic diet.

Furthermore, the group I showed improvement in glycemic profile by a significant reduction in fasting glucose and HbA1c. Similarly, Wilkinson et al.^22^ and Wei et al.^23^ showed an improvement in HbA1c and fasting glucose by IF, mainly in the subjects with higher glycemia at baseline. Also, Yamada et al.^24^ reported a significant decrease in HbA1c levels (7.6 ± 0.4% vs. 7.0 ± 0.7%) in T2DM individuals submitted to the LCD compared to those submitted to caloric restriction (7.7 ± 0.6% vs. 7.5 ± 1.0%); however, there was no significant change in body weight or BMI. In the study by Kalam et al.,^18^ although fasting glucose and HbA1c remained unchanged after six months of LCD and IF, there was a reduction in fasting insulin by 24%, which was explained by the short interventional period of IF.

In addition, the present study showed that the progression from prediabetes to T2DM was statistically higher in the control group (6.9 %) than in the IF- LCD group (2.1%). This is in accordance with the study by Wang et al.,^25^ who found a significant reduction in fasting and postprandial glucose accompanied by a reduction in the insulin dose by 8.7% in T2DM patients who underwent LCD for three months. In a meta-analysis comparing LCD with a low-fat diet in patients with T2DM, patients on LCD achieved higher diabetes remission rates at six months.^26^ This was explained by Sutton et al.,^27^ who applied IF to prediabetes and reported an increase in insulin sensitivity and beta-cell function, especially in overweight patients. Furmli et al., ^28^ also confirmed a decrease in insulin need in T2DM with IF.

Another important finding in the present study was the significantly greater reduction in LDL in group I than in group II with no change in total cholesterol, triglycerides or HDL. This was quite similar to the studies by Kalam et al.^18^ and Jacobi et al.^29^ who reported a significant reduction in LDL and total cholesterol while triglyceride and HDL levels did not change. On the other hand, four trials comparing IF to *ad libitum* food consumption found a reduction in total cholesterol with no change in LDL, triglycerides or HDL.^30^ Wilkinson et al.,^22^ however, reported a decrease from baseline in total cholesterol, LDL, and non-HDL with IF, and no change in triglycerides or HDL. Additionally, the impact of LCD was investigated and compared with a high-carb diet, with no significant changes in lipid profile and a slight reduction in triglycerides.^31^ The present study reported a significant change in albuminuria despite no change in serum creatinine, e-GFR, and incidence of nephropathy. Sulaj et al.^32^ also reported greater improvement in albuminuria with IF as compared with the Mediterranean diet and no difference in e-GFR or serum creatinine between the two groups.

Thus, regarding the primary outcomes, the incidence of unstable angina was significantly lower in group I than in controls. These results were supported by the significant elevation in Hb1Ac in group II, as well as by the significant elevation in LDL, which is the precursor of atherosclerosis. Moreover, Focardi et al.^33^ demonstrated the improvement of coronary endothelial function by LCD. Numerous studies reported the impact of IF and LCD on CVD morbidity and mortality by the enhancement of lipid and glycaemic profiles in addition to risk factor modification.^34–36^ Furthermore, the incidence of retinopathy and neuropathy was significantly lower in group I (2.1% and 2.9 %, respectively) vs. group II (6.1% and 6.9%, respectively). These outcomes proved the impact of IF and LCD on decreasing microvascular complications. In agreement with our results, Hammer et al.^37^ and Dannawi et al.^38^ revealed a reduction in retinopathy and neuropathy by IF, and Hwang et al., ^39^ revealed a recovery in microvascular endothelial function by LCD only after six weeks. A low incidence of retinopathy and neuropathy is associated with the reduction of HbA1c and fasting plasma glucose values.^40,41^

## Conclusion

IF, in combination with LCD, can play a main role in the prevention and treatment of CVD in prediabetic patients. This study showed improvement in the glycemic status and reduction in diabetes progression and a significant reduction in the incidence of retinopathy, neuropathy, and unstable angina in the IF-LCD group. In addition, the combination of IF with LCD was associated with improved primary and secondary outcomes in prediabetic patients with a decrease in cardiovascular morbidity. Increased body weight, fasting plasma glucose, HbA1c, and LDL were the independent risk factors of microvascular and macrovascular outcomes.
